# Activation of STING by SAMHD1 Deficiency Promotes PANoptosis and Enhances Efficacy of PD-L1 Blockade in Diffuse Large B-cell Lymphoma

**DOI:** 10.7150/ijbs.85236

**Published:** 2023-08-28

**Authors:** Yiqing Cai, Xiaomin Chen, Tiange Lu, Xiaosheng Fang, Mengfei Ding, Zhuoya Yu, Shunfeng Hu, Jiarui Liu, Xiangxiang Zhou, Xin Wang

**Affiliations:** 1 Department of Hematology, Shandong Provincial Hospital, Shandong University, Jinan, Shandong, 250021, China.; 2 Department of Hematology, Shandong Provincial Hospital Affiliated to Shandong First Medical University, Jinan, Shandong, 250021, China.; 3 Shandong Provincial Engineering Research Center of Lymphoma, Jinan, Shandong, 250021, China.; 4 Branch of National Clinical Research Center for Hematologic Diseases, Jinan, Shandong, 250021, China.; 5 National Clinical Research Center for Hematologic Diseases, the First Affiliated Hospital of Soochow University, Suzhou, 251006, China.

**Keywords:** DLBCL, SAMHD1, STING, PANoptosis, DMXAA.

## Abstract

Genomic instability is a significant driver of cancer. As the sensor of cytosolic DNA, the cyclic GMP-AMP synthase (cGAS)-stimulator of interferon genes (STING) pathway plays a critical role in regulating anti-tumor immunity and cell death. However, the role and regulatory mechanisms of STING in diffuse large B-cell lymphoma (DLBCL) are still undefined. In this study, we reported that sterile alpha motif and HD domain-containing protein 1 (SAMHD1) deficiency induced STING expression and inhibited tumor growth in DLBCL. High level of SAMHD1 was associated with poor prognosis in DLBCL patients. Down-regulation of SAMHD1 inhibited DLBCL cell proliferation both *in vitro* and *in vivo*. Moreover, we found that SAMHD1 deficiency induced DNA damage and promoted the expression of DNA damage adaptor STING. STING overexpression promoted the formation of Caspase 8/RIPK3/ASC, further leading to MLKL phosphorylation, Caspase 3 cleavage, and GSDME cleavage. Up-regulation of necroptotic, apoptotic, and pyroptotic effectors indicated STING-mediated PANoptosis. Finally, we demonstrated that the STING agonist, DMXAA, enhanced the efficacy of a PD-L1 inhibitor in DLBCL. Our findings highlight the important role of STING-mediated PANoptosis in restricting DLBCL progression and provide a potential strategy for enhancing the efficacy of immune checkpoint inhibitor agents in DLBCL.

## Introduction

As the most common subtype of B-cell lymphoma, diffuse large B-cell lymphoma (DLBCL) is featured by differential clinical, immunophenotypic, cytogenetic, and genetic characteristics 1. Despite the recent improvement with new treatments 2, 30%-40% of patients are insensitive to standard chemoimmunotherapy (CIT) and eventually progress to relapsed/refractory (R/R) stages 3. Patients that are resistant to CIT may benefit from other treatment options, including antibody-drug conjugates (ADC), immune checkpoint inhibitors, and chimeric antigen receptor T (CAR-T) cell therapy, all of which have been developed and incorporated into clinical practice; however, variable treatment responses are observed 4, 5. Therefore, new treatments are still urgently needed. Understanding underlying pathogenesis and identifying new biology and targets are critical for any new therapy discovery and development. Given that the malignant transformation of B-cells is correlated with the extensive DNA editing in the germinal center, targeting DNA damage response (DDR) can potentially be an important strategy for the development of new treatment for DLBCL 6, 7.

As a deoxynucleoside triphosphate hydrolase (dNTPase), sterile alpha motif and HD domain-containing protein 1 (SAMHD1) plays an important role in genomic stability by balancing the DNA precursor pools 8. In particular, SAMHD1 mainly functions by promoting the end resection process in DNA repair 9 and resolving stalled replication forks in DNA replication 10. As such, dysregulation of SAMHD1 is linked to cell cycle regulation and chemosensitivity to nucleoside analogs in human cancers 11, 12. A recent study demonstrated that targeted inhibition of SAMHD1 by hydroxyurea was a safe and effective strategy in a phase 1 trial 13, supporting an important role for SAMHD1 in hematological tumors.

It is well established that the cyclic GMP-AMP synthase (cGAS)-stimulator of interferon genes (STING) signaling pathway is critical for DNA damage perceiving 14. Induction of STING-associated inflammation facilitates the interferon (IFN)-related innate immunity priming, which is identified as a barrier to tumorigenesis 15. Interestingly, targeting DDR may induce anti-tumor immune responses via activating the cGAS-STING axis, and further enhance the efficacy of immunotherapy 16, 17. Hence, understanding the interplay between the DDR and the cGAS-STING pathway may provide a targeting strategy for the development of effective treatment for lymphoma patients 18.

In addition to immune regulation, the cGAS-STING pathway mediates cell death, including apoptosis, autophagy, necroptosis, pyroptosis, and ferroptosis 19, 20. Necroptosis and pyroptosis are the newly discovered non-apoptotic forms of cell death, characterized by the loss of cell membrane integrity and the release of cytoplasmic content 21. Necroptosis depends on the activation of mixed lineage kinase domain-like protein (MLKL), while pyroptosis relies on the cleavage of gasdermin proteins. In particular, interactions between necroptosis, apoptosis, and pyroptosis were referred to PANoptosis 22, 23. As a programmed cell death (PCD), PANoptosis depends on the PANoptosome, which contains multiple proteins essential for activating various cell death effectors 24, 25. Increasing evidence shows that PANoptosis is deeply related to tumorigenesis and immune responses 26, 27. Therefore, PANoptosis induction is considered a viable strategy in reducing tumor burden and resolving treatment resistance 28, 29. Given the importance of PANoptosis, we attempt to explore the mechanism of PANoptosis in DLBCL and hope to identify a trackable target for therapeutic interventions.

Here, we investigate the role of STING in DLBCL tumor growth and anti-DLBCL treatment. STING is activated in SAMHD1-deficient DLBCL cells, and further induces PANoptosis to suppress cell growth. Specifically, STING-mediated PANoptosis depends on the activation of MLKL, Caspase 3 (CASP3), and gasdermin E (GSDME). Moreover, we demonstrate that STING agonist DMXAA enhances programmed death ligand 1 (PD-L1) blockade efficacy. Our results highlight that targeted activation of STING holds promise in treating DLBCL.

## Materials and Methods

### Lymph node samples and peripheral blood samples

Lymph node samples were collected from 100 newly diagnosed DLBCL patients and 20 lymphoid reactive hyperplasia (RHL) patients. The diagnostic criteria were established according to the World Health Organization (WHO) classification 30. Among them, 65 enrolled DLBCL patients received treatment, of which 49 documented the treatment regimens and response (**Table S1**). Besides, the response was evaluated after every three courses of treatment, mainly based on positron emission tomography/computed tomography (PET/CT) results (**Table S1**). Peripheral blood mononuclear cells (PBMCs) of healthy donors were isolated by the Ficoll-Hypaque density gradient centrifugation method (TBD Science, Tianjin, China) and naïve CD19^+^ B-cells were separated by CD19^+^ magnetic microbeads kit (Miltenyi Biotec, Bergisch Gladbach, Germany). The purity of enriched CD19^+^ B-cells was detected by flow cytometry (FCM) analysis, as previously reported (**Figure S1A**) 31.

### CRISPR/CRISPR associated 9 (Cas9)-generated STING-knockout (KO) cells

CRISPR/Cas9 genomic editing system was applied to generate STING-KO cells. Stably expressing Cas9-gRNA was established and packaged by OBiO Technology Corp., Ltd. (Shanghai, China). The viral vector of gRNA for STING deletion was as follows: pLenti-U6-spgRNA (TMEM173)-CMV-Puro-P2A-3xFLAG-spCas9 WPRE. Vector control was annotated as WT. After 72 hours of transfection, cells were treated with 2.0μg/ml puromycin for 3 days, and single cell was screened by a limited dilution method.

### Lentiviral generation and cell transfection

Lentivirus vectors that encoded SAMHD1-knockdown (KD), SAMHD1-overexpression (LV-SAMHD1), STING-overexpression (LV-STING), and empty vectors (Ctrl and LV-Con) were constructed by GeneChem (Shanghai, China). Lentivirus infection was carried out according to the manufacturer's instruction at a multiplicity of infection (MOI) =100. Transfected cells were selected with puromycin (2.0 μg/ml) after 72 hours of transfection. Transfection efficiency was then verified by immunoblot. SAMHD1-KD, Ctrl and LV-Con sequences were available in **Table S2**.

### RNA-sequencing (RNA-seq) and bioinformatics analysis

For RNA-seq, total RNA was isolated with TRIzol reagent (Invitrogen, 15596026, CA, USA) from SAMHD1-KD LY1 cells. Novogene (Beijing, China) performed the RNA-seq experiments. Briefly, sequencing libraries were generated from purified mRNA, after which the library preparations were sequenced on an Illumina HiSeq platform and generated 150 bp paired-end reads. HTSeq v0.6.0 was then applied to calculate the numbers of reads and the fragments per kilobase million (FPKM). Gene ontology (GO), Reactome, gene set enrichment analysis (GSEA), and differential expression analyses were finally performed by R language. In addition, SAMHD1 expression in DLBCL samples was derived from the Oncomine and Cancer Genome Atlas (TCGA) databases.

### Immunofluorescence (IF)

Ctrl and SAMHD1-KD cells were mounted on glass slides, fixed in 4% paraformaldehyde, permeabilized with 0.1% Triton X-100, and then blocked with 10% goat serum in PBS. After incubating with primary antibodies, cells were probed with fluorescent secondary antibodies and stained by DAPI (Solarbio, S2110, Beijing, China). Confocal microscopic images were captured by Leica TCS SP8 MP (Leica, Wetzlar, Germany) and measured by the Image J software (National Institutes of Health, USA). The antibodies applied in IF were listed in **Table S3**.

### Neutral comet assay

Neutral comet assay was performed on Ctrl and SAMHD1-KD cells according to the manufacturer's protocol (Trevigen, Maryland, USA). DAPI (Solarbio, S2110) was used to stain the DNA fragments. Images were collected by Olympus BX51 fluorescence microscopy with ×200 objectives. Tail moments were analyzed by Open Comet software (Cambridge, USA). Fifty individual cells were counted in each experiment.

### Co-immunoprecipitation (CO-IP)

LY1 and LY3 cells transfected with LV-STING were lysed with the RIPA buffer (Beyotime, P0013, Shanghai, China). Protein lysates incubated with 3 μg of the anti-Caspase 8 (CASP8)-mouse antibody (Proteintech, 66093-1-Ig, IL, USA) or normal mouse IgG antibody (Santa Cruz, sc-2025, CA, USA) were rotated overnight at 4℃. The immune complexes were then treated with 20μl protein A/G PLUS-agarose beads (Santa Cruz, sc-2003) for 4 hours at 4℃. Beads bound to immunoprecipitates were denatured by a mental bath, followed by immunoblot analysis.

### Lactate dehydrogenase (LDH) release assay and morphology observation

According to the manufacturer's instructions, a CytoTox 96 non-radioactive cytotoxicity assay kit (Promega, G1780, Madison, USA) was performed to test supernatant LDH levels of cells. For morphological investigation, DLBCL cells transfected with lentivirus vectors were visualized by the OLYMPUS CKX41 inverted microscope at ×200 objectives in three different fields. Image processing was performed by Image J software.

### Immunohistochemistry (IHC)

IHC staining was performed as previously introduced 31. The number of positive cells referred to the expression level. The receiver operating characteristic (ROC) curve was constructed by the numbers of positive cells, of which the Youden's index was recognized as the evaluation standard (value = 0.337). With these criteria, tissues were interpreted as “positive (positive rate > 33.7%)” or “negative (positive rate ≤ 33.7%).” The antibody applied in IHC was Rabbit anti-SAMHD1 (1:200, Proteintech, 12586-1-AP).

### Cell lines and reagents

Human DLBCL cell lines LY1, LY3, and LY8 were cultured in Iscove's modified Dulbecco's medium (IMDM) (Gibco, CA, USA) supplemented with 10% heat-inactivated fetal bovine serum (HyClone, UT, USA). Cells were left to incubate at 37°C in a humidified 5% CO_2_ atmosphere. DMXAA (S1537) and BMS1166 (S8859) were obtained from Selleckchem (TX, USA). CalcuSyn software (Cambridge, UK) was applied to evaluate the effectiveness of drug combinations, representing by the combination index (CI) values. In particular, CI < 1 indicates synergism, CI=1 shows an additive effect, and CI > 1 represents antagonism.

### Cell viability detection

Cell viability was measured by the cell counting kit-8 (CCK-8) assay (Dojindo, CK04, MD, USA). In briefly, cells were seeded in 96-well plates at 1×10^4^ cells/well and stained with 10μl CCK-8/well at a certain point in time, after which optical density was detected at 450nm by Multiskan GO Microplate Reader (Thermo Scientific, Rockford, IL, USA).

### Western blotting (WB)

WB was performed as previously introduced 32. Cell lysates were obtained using the RIPA buffer (Beyotime, P0013B) mixed with phosphatase inhibitor cocktail (PhosSTOP, Roche, Basel, Switzerland). Total proteins were loaded in the SDS-PAGE (Bio‐Rad, California, USA) and transferred to polyvinylidene fluoride (PVDF) membranes. After blocking with 10% skimmed milk, PVDF membranes were incubated with primary and secondary antibodies. The antibodies were listed in **Table S4**.

### FCM

Apoptosis rates were detected by Annexin V-PE/7AAD or Annexin V-FITC/PI double-staining FCM (BD Biosciences, Bedford, MA, USA). The percentage of apoptotic cells was conducted on Navios Flow Cytometer (Beckman Coulter, CA, USA).

### *In vivo* mice xenograft models

Animal experimental procedures were performed in accordance with protocols approved by the Institutional Animal Care and Research Advisory Committee of Shandong Provincial Hospital. Mice were purchased from the Vital River Laboratory Animal Technology Co., Ltd. (Beijing, China). For the *in vivo* study of SAMHD1, a total of 1×10^7^ Ctrl or SAMHD1-KD LY1 cells were subcutaneously injected into the right upper flanks of severe combined immunodeficiency (SCID) beige mice (n = 6/group), respectively. For the efficacy of drug combination studies, xenograft models were established in 4-week-old female Balb/c nude mice by injecting 1×10^7^ wild-type LY1 cells into the subcutaneous of right lower flanks. When tumors reached 100 mm^3^, tumor-bearing mice were randomized into specified groups (n = 6/group). For monotherapy, the mice were intra-peritoneally injected with 100μl BMS1166 (250 μg/ml) or DMXAA (20 mg/kg). For combination therapy, BMS1166 (250 μg/ml) and DMXAA (20 mg/kg) were alternately injected every other day. Tumor dimensions were measured every 2 days, which were calculated using the equation *V* = (*l* × *w*^2^) × 0.5. Mice were sacrificed until any one of several criteria were met, including severe lethargy, more than 10% body-weight loss, and approximately 2cm of tumor diameter.

### Statistical analysis

Data was presented as mean ± standard deviation (SD) from at least three separate experiments. All statistical analyses were performed with SPSS Statistics version 20.0 and the GraphPad Prism software (version 8.0a, La Jolla, CA, USA). Statistical significance between the two groups was determined by unpaired two-tailed t-test with assumed normal distribution. If normality tests failed, Mann-Whitney tests were applied. Three or more groups were analyzed by Welch's one-way ANOVA analysis with Dunnett's T3 tests. Proliferation curves were measured by two-way ANOVA analysis with Sidak correction. Log-rank tests were used in survival analysis. Contingency tables were determined by two-tailed χ^2^ test. P-value < 0.05 was considered as statistical significance, including ^*^p < 0.05, ^**^p < 0.01, ^***^p < 0.001, and ^****^p < 0.0001.

## Results

### SAMHD1 is highly expressed in DLBCL and associated with poor prognosis of patients

**Figure 1:**
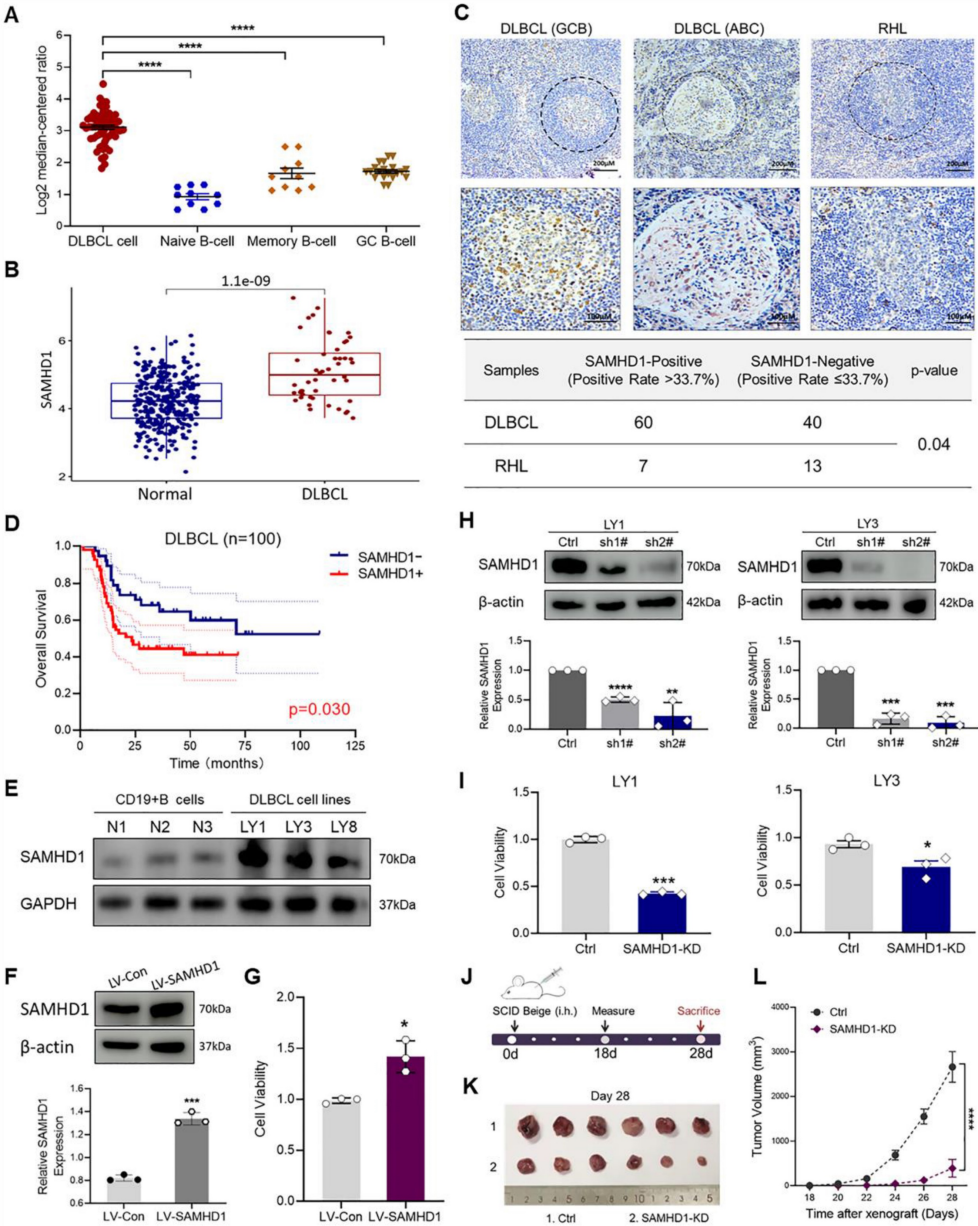
** SAMHD1 expression is up-regulated in DLBCL and related to tumor growth. A, B.** The mRNA levels of SAMHD1 in DLBCL samples from the Oncomine (**A**) and TCGA database (**B**). **C.** IHC staining revealed the expression of SAMHD1 in GCB-like DLBCL, ABC-like DLBCL, and RHL tissues (upper), followed by relative quantitative analysis (lower, p=0.04). P value came from the Chi-square test. **D.** Kaplan Meier plots for DLBCL patients enrolled in IHC staining (n=100, p=0.03). **E.** Protein levels of SAMHD1 in normal CD19^+^ B-cells (N1, N2, N3) and DLBCL cell lines (LY1, LY3, LY8) were detected by western blotting. **F, G.** LY1 cells were transfected with LV-Con or LV-SAMHD1 sequences. After stable transfection, transfection efficiency was examined by immunoblot (**F**), while cell viability was detected by CCK-8 assay (**G**). **H, I**. LY1 and LY3 cells were transfected with Ctrl or SAMHD1-KD sequences (sh1#, and sh2#). After stable transfection, knockdown efficiency was examined by immunoblot (**H**), while cell viability was detected by CCK-8 assay (**I**). **J-L.** Schematic of *in vivo* tumor growth investigation (**J**). SCID beige mice were injected with Ctrl or SAMHD1-KD LY1 cells (n=6/group). Tumor bodies were taken on day 28 (**K**). Tumor growth curves were shown from day 18 to day 28 (**L**). Immunoblot images in **E, F**, and **H** were the representation of 3 independent experiments. Vertical bars indicated mean ± SD. P values came from Kruskal-Wallis test followed by Dunn's test (**A**), Log-rank test (**D**), unpaired two-tailed t-test (**B, F, G, H, I**), and Two-way ANOVA with Sidak correction (**L**). ^*^p < 0.05, ^**^p < 0.01, ^***^p < 0.001, and ^****^p < 0.0001.

We first sought to determine the expression of SAMHD1 in DLBCL patients. Analyses of the Oncomine and TCGA databases revealed a significant increase of SAMHD1 mRNA in DLBCL tissues, compared with normal B-cell subtypes (**Figure 1A, B**). Subsequently, the protein levels of SAMHD1 in DLBCL tissues were determined by IHC in 100 DLBCL patients. Specifically, the positive expression of SAMHD1 was significantly higher in DLBCL patients (positive rate = 60.0%, 60/100) than that in RHL tissues (positive rate = 35.0%, 7/20) (**Figure 1C**). In addition, SAMHD1 expression showed no difference in DLBCL samples with different cell-of-origin (COO) (**Figure 1C**).

To further investigate the clinical significance of SAMHD1 expression in DLBCL patients, we analyzed the clinical information of patients enrolled in IHC staining (**Table 1**). Interestingly, SAMHD1-positive patients were featured with defective B symptoms and decreased serum LDH (**Table 1**). Although SAMHD1 was associated with antiviral infection, SAMHD1 expression showed no correlation with Epstein-Barr virus (EBV) or Hepatitis B virus (HBV) infections in DLBCL patients (**Table 1**). Analysis of clinical treatment responses was also performed to identify the effects of SAMHD1 on therapeutic efficacy. Forty-nine enrolled patients received systematic treatment and effect evaluation, and 71.4% of them (10/14) achieved complete remission (CR) after treatment with six cycles of rituximab, cyclophosphamide, doxorubicin, vincristine, and oral prednisone (R-CHOP) (**Table S1**). However, differential expression of SAMHD1 showed no significant impact on clinical efficacy (**Table 1**). Subsequent survival analysis revealed a poorer prognosis in SAMHD1-positive DLBCL patients (**Figure 1D**), indicating that high SAMHD1 expression led to inferior prognosis in DLBCL patients.

### SAMHD1 expression is important for DLBCL cell growth

After establishing the correlation between the SAMHD1 expression and clinical outcomes of DLBCL patients, we attempted to explore the role of SAMHD1 in tumorigenesis. To select proper cell models, immunoblot analysis was used to determine the protein levels of SAMHD1 in DLBCL cell lines. As shown in **Figure 1E**, SAMHD1 expression was increased in several DLBCL cell lines, especially in LY1 and LY3 cells. To verify whether SAMHD1 expression contributed to cell survival, we constructed SAMHD1-overexpressed LY1 cells utilizing the LV-SAMHD1 sequence (**Figure 1F).** As expected, cell viability was increased with SAMHD1 overexpression (**Figure 1G**). To confirm that these findings were due to SAMHD1 expression, SAMHD1-KD models were established in LY1 and LY3 cells. Individual shRNA sequence sh2# with significant knockdown effects was selected for the functional analysis (**Figure 1H**). In contrast to SAMHD1 overexpression, SAMHD1 deficiency significantly suppressed cell viability (**Figure 1I**).

To further investigate the role of SAMHD1 *in vivo*, we examined the tumor growth in a xenograft mouse model established by Ctrl and SAMHD1-KD LY1 cells (**Figure 1J**). SAMHD1 deficiency markedly reduced tumor size and suppressed tumor growth (**Figure 1K, L**).

### SAMHD1 deficiency induces DNA damage and double-strand DNA (dsDNA) accumulation

Next, we sought to explore the mechanism by which SAMHD1 deficiency suppressed DLBCL tumor growth. Our analysis with FCM detected a significant increase in apoptosis rates in SAMHD1-KD DLBCL cells (**Figure S2A**). Additionally, optical microscopy revealed marked morphological changes in these cells, including cell shrinkage and cell membrane swelling (**Figure S2B**). Morphological changes suggested the impairment of cell membrane integrity in SAMHD1-KD DLBCL cells, which was confirmed by the high levels of supernatant LDH (**Figure S2C**). Given the specific changes in phenotypes, we hypothesized that down-regulation of SAMHD1 in DLBCL cells led to cell death.

**Figure 2:**
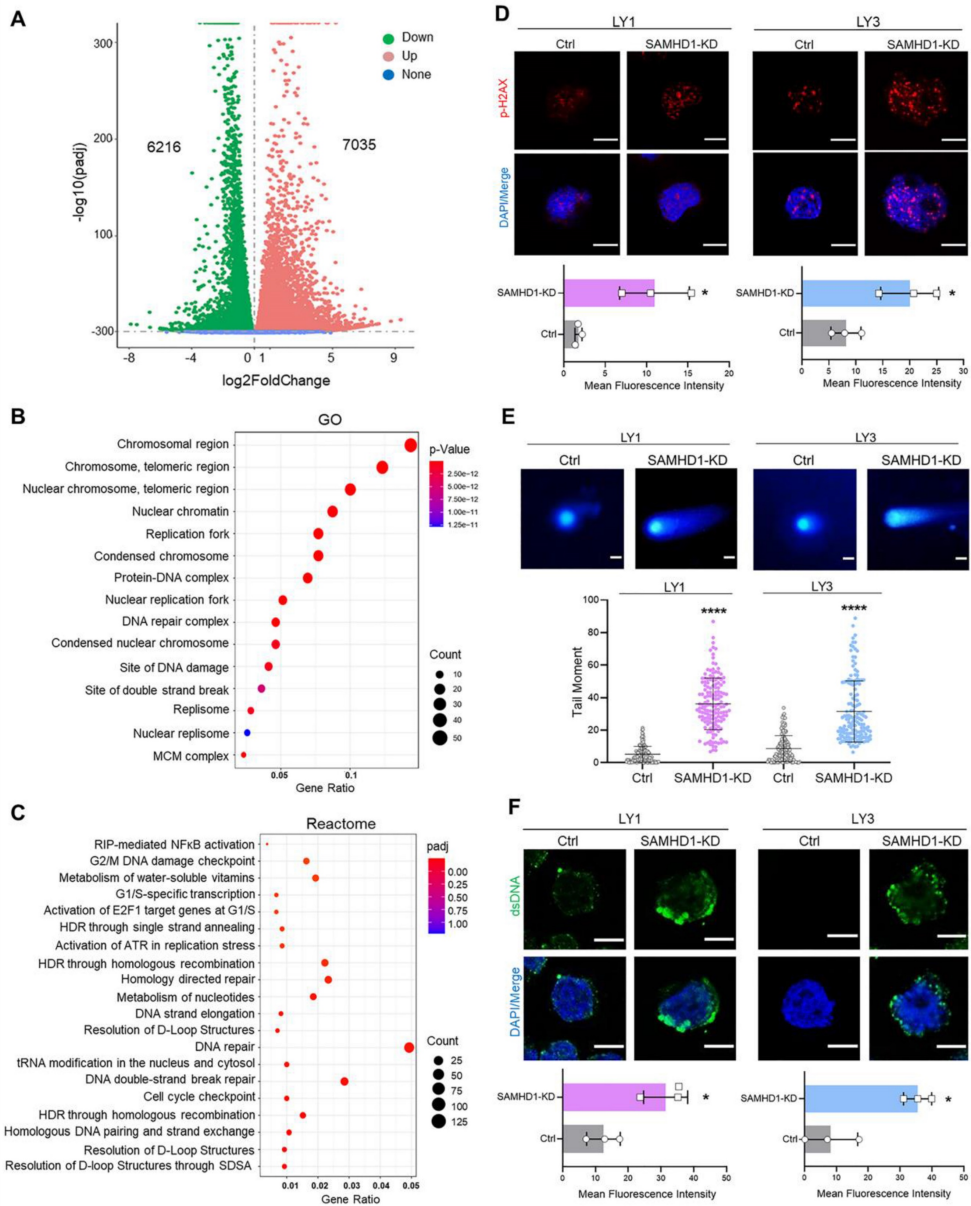
** SAMHD1 deficiency induces DNA damage and dsDNA accumulation. A-C.** RNA-seq was performed in Ctrl and SAMHD1-KD LY1 cells (three biological replicates for each group). Volcano plot revealed DEGs in SAMHD1-KD cells compared with Ctrl cells (**A**). Bubble plots revealed the significantly up-regulated GO terms (**B**) and Reactome pathways (**C**) in SAMHD1-KD LY1 cells.** D.** Fluorescence plots (upper) and fluorescence density (lower) of H2AX in Ctrl and SAMHD1-KD DLBCL cells. Scale bar=10μm. **E.** Representative images (upper) and qualification (lower) of neutral comet assay revealed the abundance of DNA fragments in Ctrl and SAMHD1-KD DLBCL cells. Scale bar=20μm. Individual dots represented single cells. **F.** Fluorescence plots (upper) and fluorescence density (lower) of dsDNA in Ctrl and SAMHD1-KD DLBCL cells. Scale bar=10μm. Vertical bars indicated mean ± SD. P values from unpaired two-tailed t-test (**D, E, F**). ^*^p<0.05, ^****^p<0.0001.

To further explore the underlying mechanisms of SAMHD1-mediated cell death, we performed RNA-seq in SAMHD1-deficient LY1 cells and screened the differentially expressed genes (DEGs) (**Figure 2A**). GO analysis displayed that DEGs were enriched in the chromosomal regions, nuclear chromatin, replication forks, DNA damage, and double-strand break (DSB) sites (**Figure 2B**). Reactome analysis further identified the enrichment in biochemical reactions related to DNA damage repair, mainly including DSB repair, homologous recombination repair, nucleotide metabolism, and cell cycle checkpoints (**Figure 2C**).

To verify the generation of DNA damage, we investigated the density of DNA damage in SAMHD1-deficient DLBCL cells. H2AX and Hsp60, the DNA damage markers, were used to locate damage sites. Of note, down-regulation of SAMHD1 dramatically increased the intensity of p-H2AX in the nucleus (**Figure 2D**); however, the intensity of cytosolic Hsp60 showed no significant changes (**Figure S3A**). Along with DNA damage, neutral comet assay detected the extended tails in SAMHD1-deficent cells (**Figure 2E**), suggesting the accumulation of DNA fragments. Besides, cytosolic dsDNA was increased in these cells (**Figure 2F**). Thus, SAMHD1 deficiency leads to DNA damage and dsDNA accumulation in DLBCL cells.

### Down-regulation of SAMHD1 promotes the expression of the STING-related DNA-sensing pathway

**Figure 3:**
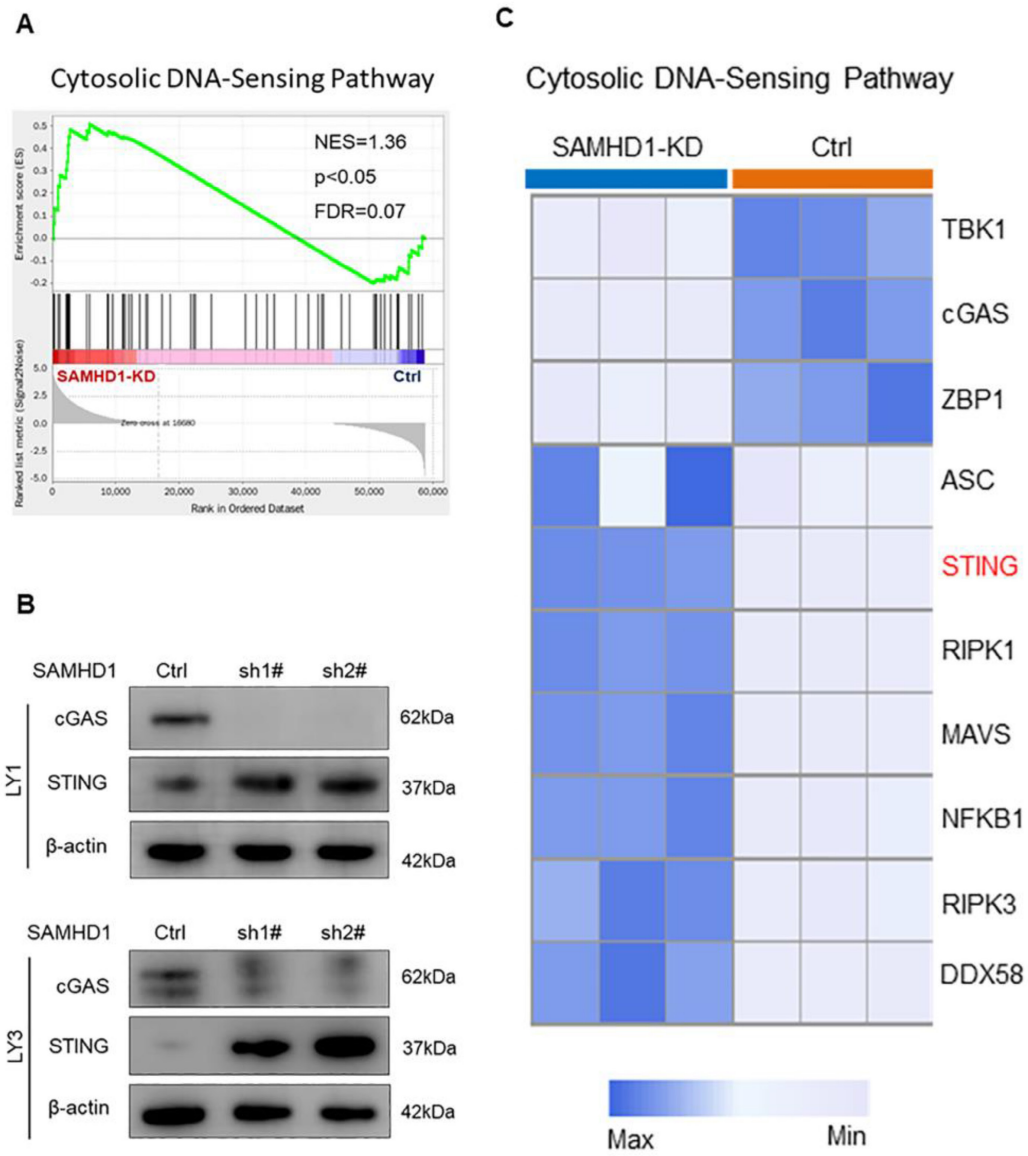
** Down-regulation of SAMHD1 promotes the expression of the STING-related DNA-sensing pathway. A.** GSEA plot of **t**he cytosolic DNA-sensing pathway (NES=1.36, p<0.05, FDR=0.07). **B.** Immunoblot showed the protein levels of cGAS and STING proteins in Ctrl and SAMHD1-KD DLBCL cells. Immunoblot images were the representation of 3 independent experiments. **C.** Heatmap revealed the DEGs in** t**he cytosolic DNA-sensing pathway between Ctrl and SAMHD1-KD LY1 cells.

As SAMHD1 deficiency induced DNA damage, we further examined the expression of DNA-sensing pathways in SAMHD1-deficient cells. GSEA analysis revealed the up-regulation of the STING-mediated cytosolic DNA-sensing pathway in SAMHD1-KD LY1 cells (p<0.05; **Figure 3A**). However, immunoblot analysis verified that SAMHD1 deficiency mainly promoted the expression of STING, rather than cGAS (**Figure 3B**). To explore the biological functions of SAMHD1-KD-induced STING, we analyzed the DEGs in the cytosolic DNA-sensing pathway. Although STING was involved in innate immune activation 33, IFN regulators were inactivated in SAMHD1-KD DLBCL cells, including TANK-binding kinase 1 (TBK1) and interferon regulatory factor 3 (IRF3) (**Figure 3C, Figure S4A**). Interestingly, SAMHD1 deficiency promoted the transcription of genes related to cell death, including receptor-interacting protein kinase 1 (RIPK1), receptor-interacting protein kinase 3 (RIPK3), nuclear factor kappa-B (NF-κB), and apoptosis-associated speck-like protein containing CARD (ASC) (Log2FoldChange > 2, p < 0.05; **Figure 3C**). These data suggest the correlation between STING and cell death regulation.

### STING activation induces multiple forms of cell death to suppress DLBCL cell growth

**Figure 4:**
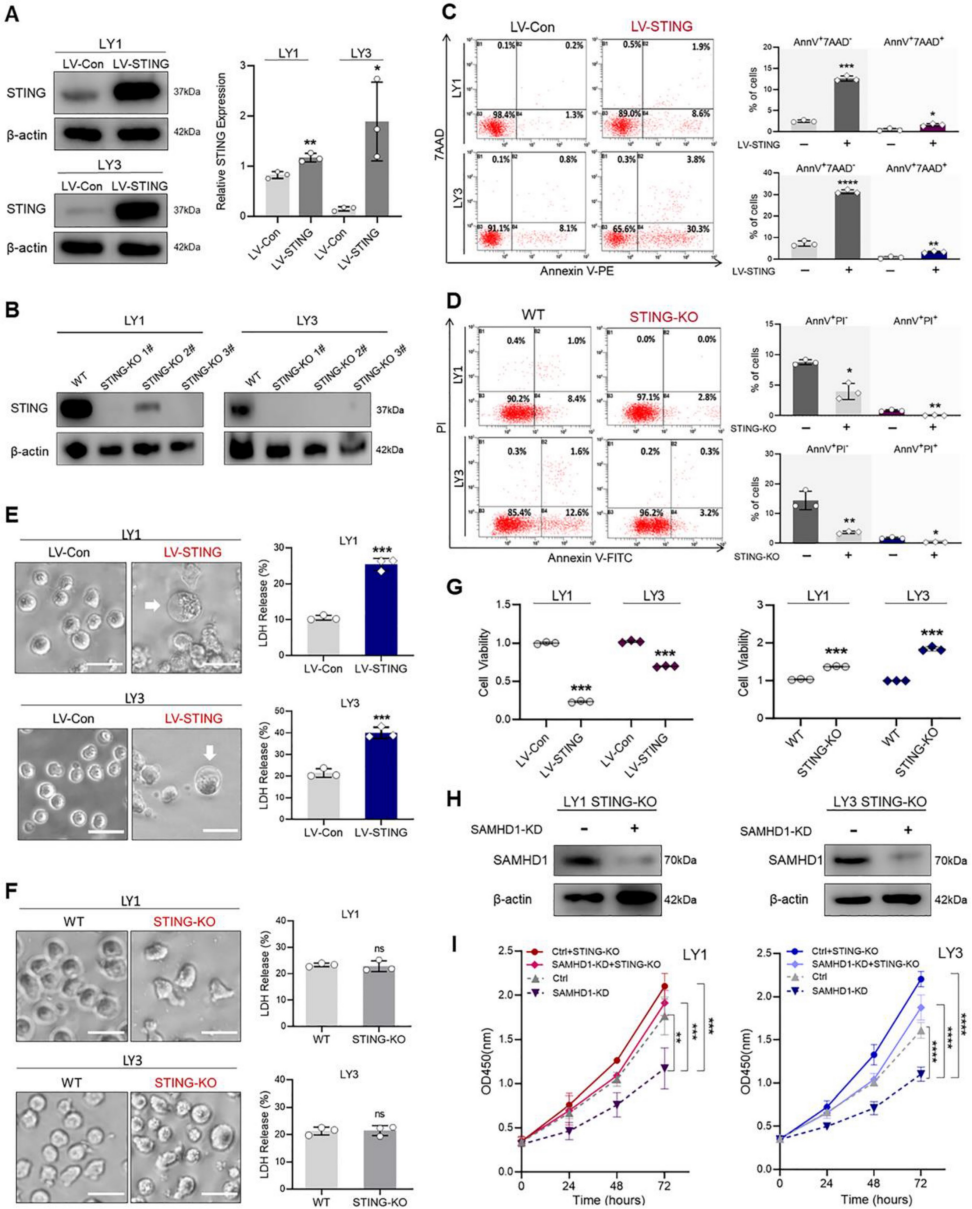
** STING activation induces multiple forms of cell death to suppress DLBCL cell growth. A**. STING overexpression model was established in LY1 and LY3 cells utilizing LV-STING sequences. Transfection efficiency was determined by immunoblot. **B.** STING-KO model was constructed in LY1 and LY3 cells by transfecting three independent CRISPR/Cas9-mediated STING-KO sequences (STING-KO #1, #2, and #3). Transfection efficiency was determined by immunoblot. **C.** Annexin V-PE/7AAD double staining flow cytometry revealed the scatter plots (left) and quantitative apoptosis rates (right) in LV-Con and LV-STING DLBCL cells. **D.** Annexin V-FITC/PI double staining flow cytometry revealed the scatter plots (left) and quantitative apoptosis rates (right) in WT and STING-KO DLBCL cells.** E**. Microscopic images (left, scale bar=50μm) and supernatant LDH levels (right) of LV-Con and LV-STING DLBCL cells. White arrows indicated cell membrane swelling.** F**. Microscopic images (left, scale bar=50μm) and supernatant LDH levels (right) of WT and STING-KO DLBCL cells. **G.** CCK-8 assay revealed the cell viability of LV-STING (left) and STING-KO (right) DLBCL cells, which were compared with empty vectors. **H, I.** Ctrl and SAMHD1-KD sequences were transfected in STING-KO DLBCL cells. Transfection efficiency was determined by immunoblot (**H).** Cell proliferation of Ctrl, SAMHD1-KD, Ctrl+STING-KO, and SAMHD1-KD+STING-KO DLBCL cells was compared by CCK-8 assay (**I**). Immunoblot images in **A, B,** and **H** were the representation of 3 independent experiments. Vertical bars indicated mean ± SD. P values from unpaired two-tailed t-test (**A, C, D, E, F,** and** G**) and Two-way ANOVA with Sidak correction (**I**). ^*^p<0.05, ^**^p<0.01, ^***^p<0.001, ^****^p<0.0001, and ns=no significance.

To investigate whether cell death was mediated by STING, we constructed lentivirus-mediated LV-STING and CRISPR/Cas9-mediated STING-KO models in LY1 and LY3 cells, respectively (**Figure 4A, B**). As shown in the FCM analysis, STING overexpression significantly increased the percentage of Annexin V^+^/7AAD^-^ and Annexin V^+^/7AAD^+^ cells (**Figure 4C**). In contrast, Annexin V-FITC^+^/PI^-^ and Annexin V-FITC^+^/PI^+^ cells were decreased by STING deletion (**Figure 4D**). These results were indicative of STING activation-induced cell apoptosis. Subsequently, morphological changes and LDH release were detected to verify the generation of non-apoptotic cell death. It was worth noting that LV-STING cells, rather than STING-KO cells, were featured with cell membrane swelling and high levels of supernatant LDH (**Figure 4E**, **F**).

Given the functions of STING in cell death induction, we further explored whether STING overexpression suppressed DLBCL cell growth. As expected, STING overexpression decreased cell viability, while STING deletion enhanced cell viability (**Figure 4G**). To better understand whether STING was involved in SAMHD1-mediated cell survival, intrinsic STING was deleted in DLBCL cells with and without SAMHD1-KD (**Figure 4H**). Genetic deletion of STING in SAMHD1-KD cells restored cell survival to a similar level of Ctrl cells, suggesting that STING mediated SAMHD1 deficiency-induced cell death (**Figure 4I**). Together we demonstrate the functions of STING in DLBCL, that is, up-regulation of STING suppresses DLBCL cell growth by inducing various forms of cell death.

### STING activates MLKL, CASP3, and GSDME to induce PANoptosis

**Figure 5:**
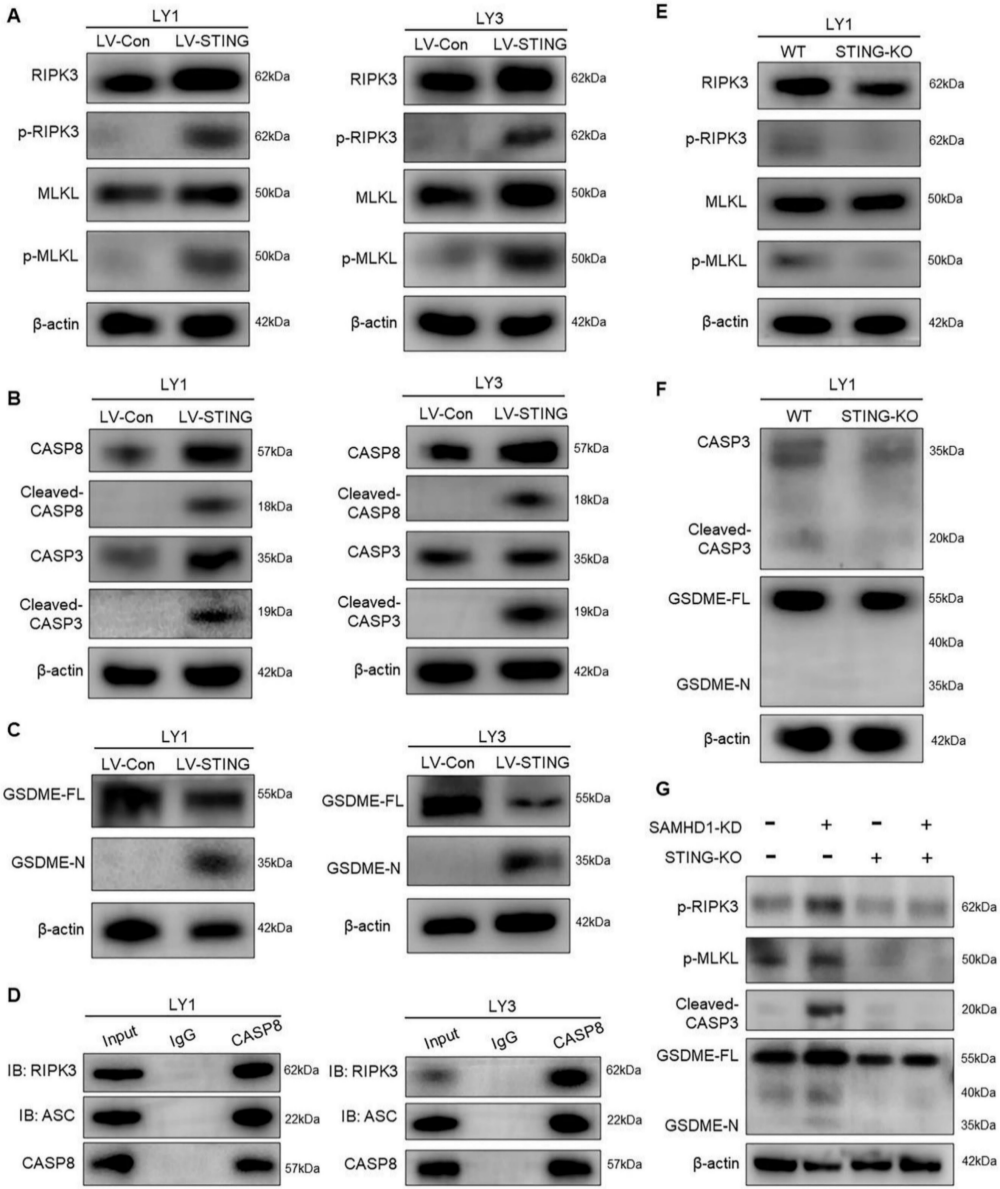
** STING activates MLKL, CASP3, and GSDME to induce PANoptosis.A-C.** Immunoblot revealed the expression of different cell death effectors in LV-Con and LV-STING DLBCL cells, including the effectors of necroptosis (RIPK3, p-RIPK3, MLKL, and p-MLKL) (**A**), apoptosis (CASP8, cleaved-CASP8, CASP3, and cleaved-CASP3) (**B**), and pyroptosis (GSDME-FL and GSDME-N) (**C**). **D.** Interactions between CASP8, RIPK3, and ASC in LV-STING DLBCL cells were detected by CO-IP assay. **E, F**. Immunoblot revealed the expression of cell death effectors in WT and STING-KO DLBCL cells, including the effectors of necroptosis (RIPK3, p-RIPK3, MLKL, and p-MLKL) (**E**), apoptosis and pyroptosis (CASP3, cleaved-CASP3, GSDME-FL, and GSDME-N) (**F**)**. G.** Immunoblot revealed the protein levels of p-RIPK3, p-MLKL, cleaved-CASP3, and GSDME-N in LY1 cells transfected with Ctrl, SAMHD1-KD, Ctrl+STING-KO, and SAMHD1-KD+STING-KO. Immunoblot images were the representation of 3 independent experiments.

Next, we attempted to explore the underlying mechanisms of STING-induced cell death. STING is critical in driving PCD, including necroptosis, apoptosis, and pyroptosis 34. To determine the modes of STING-induced cell death, we performed immunoblot analysis to verify the downstream pathways in LV-STING DLBCL cells. RNA-seq analyses previously revealed an increase in RIPK1 and RIPK3, the major regulators of necroptosis 35, 36. Consistently, phosphorylated RIPK3 and MLKL were increased in DLBCL cells with overexpressed STING, indicative of necroptosis induction (**Figure 5A**). In addition to necroptosis, we also detected caspase-dependent apoptosis and pyroptosis. Of note, STING overexpression promoted the activation of apoptotic effectors, represented by the cleavage of CASP8 and CASP3 (**Figure 5B**). Pyroptosis is a non-apoptotic cell death that relies on caspase-mediated gasdermin cleavage. As the specific gasdermin protein in cancer cells, GSDME responds to CASP3 and further induces secondary pyroptotic cell death 37. With CASP3 activation, GSDME proteins were cleaved in LV-STING DLBCL cells, indicated by the expression of GSDME-N fragments (**Figure 5C**).

Interestingly, interactions between necroptosis, apoptosis, and pyroptosis lead to the appearance of PANoptosis. Recent studies demonstrated that CASP8 interacted with RIPK3 and ASC might function as a cell death signaling scaffold to induce PANoptosis 38, 39. CO-IP assay also revealed that CASP8 directly interacted with RIPK3 and ASC in LV-STING DLBCL cells, which provided the foundation for cell death induction (**Figure 5D**). Collectively, the above results revealed the importance of STING in PANoptosis induction.

To verify whether PANoptosis concurrently responded to STING activation, we detected the expression of cell death effectors in STING-KO DLBCL cells. As shown in **Figure 5E**, protein levels of phosphorylated RIPK3 and MLKL were reduced by STING deletion. Besides, cleaved-CASP3 and GSDME-N fragments showed no significance between WT and STING-KO cells (**Figure 5F**).

As SAMHD1 deficiency led to STING activation, we further explored whether SAMHD1 deficiency induced PANoptosis and, if so, whether STING was the mediator. Consistent with what was observed in STING overexpression, down-regulation of SAMHD1 increased phosphorylated RIPK3, phosphorylated MLKL, cleaved-CASP3, and GSDME-N fragments (**Figure 5G**). STING deletion conversely decreased the expression of cell death effectors in these SAMHD1-deficient cells (**Figure 5G**). These data establish that SAMHD1 deficiency promotes STING activation to induce PANoptosis in DLBCL.

### DMXAA inhibits DLBCL cell growth by inducing cell death

**Figure 6:**
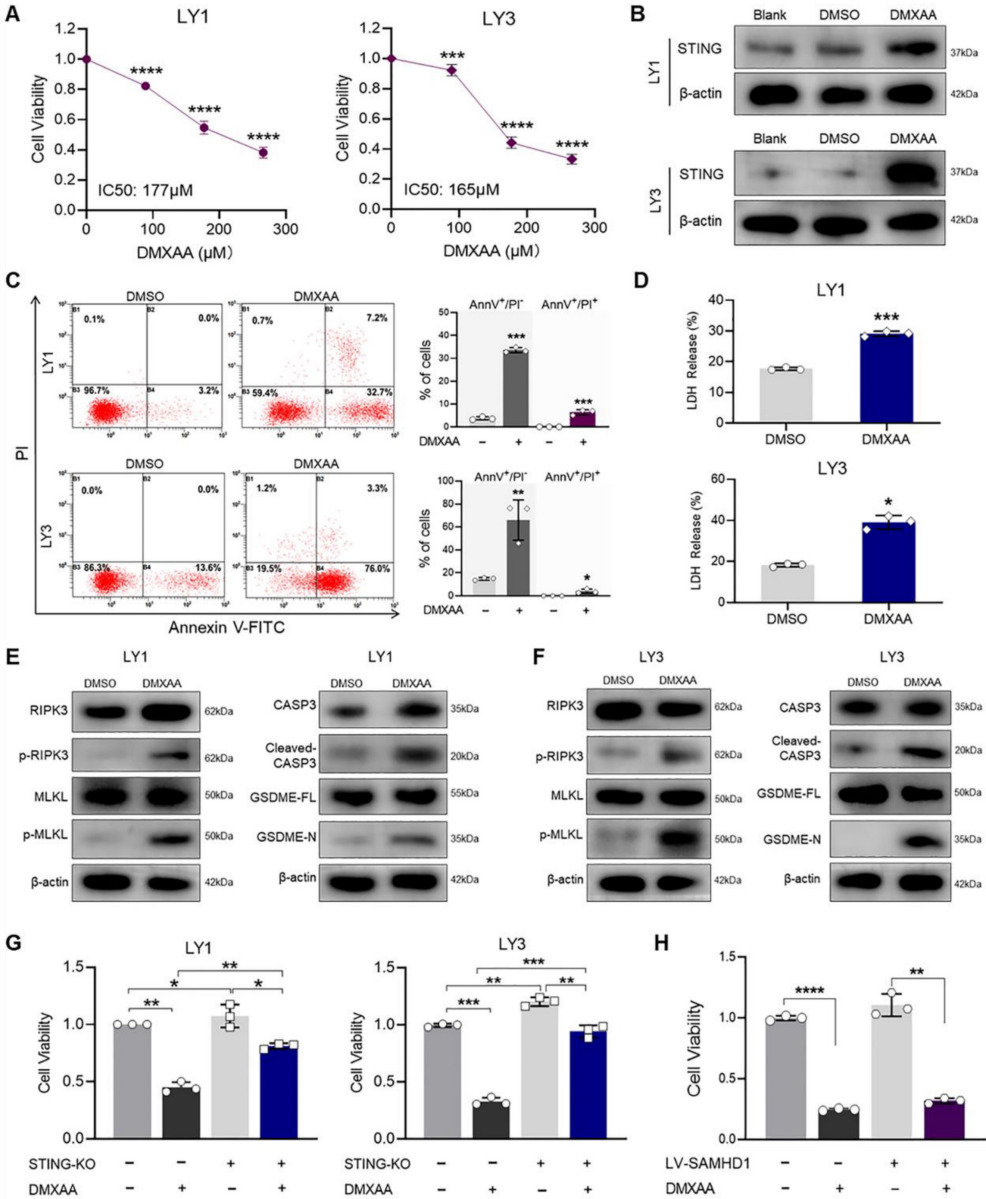
** DMXAA inhibits DLBCL cell growth by inducing cell death. A.** LY1 and LY3 cells were treated with the concentration gradients of DMXAA for 24 hours. CCK-8 assay revealed the cell viability and IC50 value in DMXAA-treated cells (LY1 IC50=177μM, LY3 IC50=165μM). **B-F**. LY1 and LY3 cells were treated with DMSO or 177μM DMXAA for 24 hours. STING expression was measured by immunoblot analysis. Wild-type cells without treatment were the blank control (Blank) (**B**). Scatter plots (left) and quantitative apoptosis rates (right) were revealed by Annexin V-FITC/PI double staining flow cytometry (**C**). Supernatant LDH levels were detected by LDH release assay (**D**). Immunoblot showed the protein levels of cell death effectors in LY1 (**E**) and LY3 (**F**) cells.** G.** WT and STING-KO DLBCL cells were treated with DMSO or 177μM DMXAA for 24 hours. Cell viability was determined by CCK-8 assay. **H.** LV-Con and LV-SAMHD1 LY1 cells were treated with DMSO or 177μM DMXAA for 24 hours. Cell viability was determined by CCK-8 assay. Vertical bars indicated mean ± SD. P values from unpaired two-tailed t-test (**A, C, D, F, G, H**). ^*^p<0.05, ^**^p < 0.01, ^***^p < 0.001, and ^****^p < 0.0001.

After showing that STING activation contributed to suppressing DLBCL tumor growth, we tested the anti-tumor effects of STING agonists in these cells. DMXAA is a STING agonist with potential anti-tumor activity 40, 41. CCK-8 assay showed that DMXAA treatment decreased DLBCL cell viability in a dose-dependent manner (**Figure 6A**). To explore the mechanisms of DMXAA, we detected phenotypic changes and cell death effectors in DMXAA-treated DLBCL cells. As expected, DMXAA promoted STING expression in DLBCL cells (**Figure 6B**), consistent with the increase in apoptosis rates and LDH release levels (**Figure 6C, D**). Immunoblot further verified the activation of cell death effectors, represented by the expression of p-RIPK3, p-MLKL, cleaved-CASP3, and GSDME-N (**Figure 6E, F**).

To investigate whether the inhibitory effects of DMXAA were dependent on STING, we treated STING-KO DLBCL cells with DMXAA. Genetic deletion of STING significantly decreased the inhibition of DMXAA on cell viability (**Figure 6G**). As STING expression could be promoted by SAMHD1 deficiency, we further examined the effects of DMXAA on LV-SAMHD1 LY1 cells. Of note, cell viability of SAMHD1-overexpressed cells was significantly suppressed by DMXAA treatment, suggesting that STING activation could overcome SAMHD1-mediated tumor growth (**Figure 6H**). Together our findings suggest the potential role of STING agonists in anti-DLBCL treatment.

### DMXAA enhances the efficacy of BMS1166 in DLBCL

**Figure 7:**
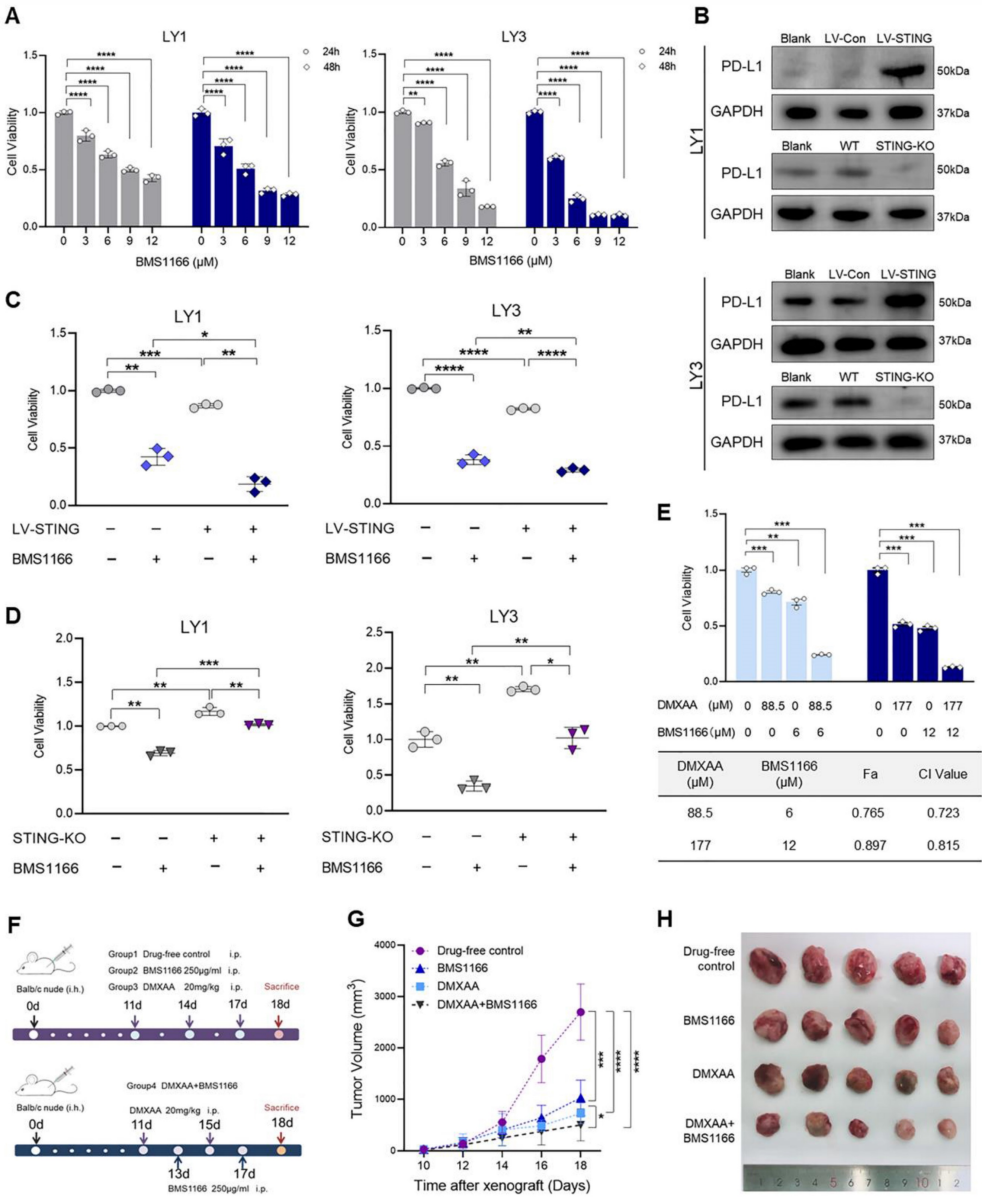
** DMXAA enhances the efficacy of BMS1166 in DLBCL. A.** LY1 and LY3 cells were treated with DMSO or BMS1166 (3, 6, 9, 12μM) for 24 or 48 hours. Cell viability was detected by CCK-8 assay. **B.** Immunoblot revealed the expression of PD-L1 proteins in LV-Con, LV-STING, WT, and STING-KO DLBCL cells. Wild-type cells without treatment were the blank control (Blank).** C, D.** DLBCL cells transfected with LV-Con, LV-STING, WT, and STING-KO were treated with DMSO or 9μM BMS1166 for 24 hours. Cell viabilities of LV-STING (**C**) and STING-KO (**D**) groups were determined by CCK-8 assay. **E.** LY1 cells were treated with DMXAA and BMS1166 at a concentration ratio of 88.5:6. Cell viability (upper) and CI values (lower) of different regiments were presented after 24 hours of incubation**. F-H.** Balb/c nude mice were injected with LY1 cells and randomized into 4 groups. Groups 1-3 were set for monotherapy, where mice were injected with drug-free control or 250μg/ml BMS1166 or 20mg/kg DMXAA every two days. Group 4 was applied for drug combination, where mice were alternately injected with 250μg/ml BMS1166 and 20mg/kg DMXAA every other day (**F**). Tumor volumes were measured every other day from day 10 (**G**). Images of tumor bodies were taken on day 18 (**H**, n=5/group, scale bar=1cm). Immunoblot images were the representation of 3 independent experiments. Vertical bars indicated mean ± SD. P values from unpaired two-tailed t-test (**C, D**), Welch's one-way ANOVA test with Dunnett's T3 test (**E**), and Two-way ANOVA analysis with Sidak correction (**A, G**). *p<0.05, ^**^p<0.01, ^***^p<0.001, and ^****^p<0.0001.

The potential of STING agonists alone or, more importantly, in combination with immune checkpoint inhibitors draws the great interest of both clinical and pre-clinical research. For example, STING activation can overcome resistance to programmed death 1 (PD-1)/PD-L1 blockade 42. Given that PD-L1 expression was associated with poor overall survival of DLBCL patients 43, we investigated the efficacy of PD-L1 blockade in DLBCL cells. As shown in **Figure 7A**, BMS1166, a novel small molecular inhibitor, suppressed cell proliferation in a dose- and time-dependent manner. STING activation was previously demonstrated to boost PD-L1 expression in tumor cells 44. We also found that the protein levels of PD-L1 were increased by LV-STING and diminished by STING-KO in DLBCL cells (**Figure 7B**). High levels of PD-L1 were related to high sensitivity to PD-L1 blockade. Notably, BMS1166 treatment significantly reduced the viability of LV-STING DLBCL cells and, to a less degree, decreased the viability of the STING-KO DLBCL cells (**Figure 7C, D**).

Previous studies illustrated that PD-L1 expression was higher in the activated B-cell (ABC) subtype compared with germinal center B-cell (GCB) subtype 43, 45. Drug combination investigations were used to address whether the efficacy of PD-L1 blockade could be improved in PD-L1^low^ GCB-like DLBCL. Concentrations below or equal to the IC50 of different agents were used in different regimens (**Figure S5A**). As shown in **Figure 7E**, drug combination dramatically decreased the viability of LY1 cells compared to monotherapy. In addition, the CI value was less than 0.8 in a low-dose combination, representing the synergetic effects (**Figure 7E**).

A cell-derived xenograft (CDX) model was then constructed to investigate the efficacy of the drug combination *in vivo* (**Figure 7F**), where DMXAA and BMS1166 functioned at the effective concentrations 46. Compared with BMS1166 monotherapy, combination treatment significantly suppressed the growth of GCB-like DLBCL cells (**Figure 7G, H**). Moreover, DMXAA treatment could induce coagulative necrosis in primary tumor lesions (**Figure 7H**). Collectively, these results support that STING agonist DMXAA enhances the efficacy of PD-L1 blockade in DLBCL.

## Discussion

**Figure 8:**
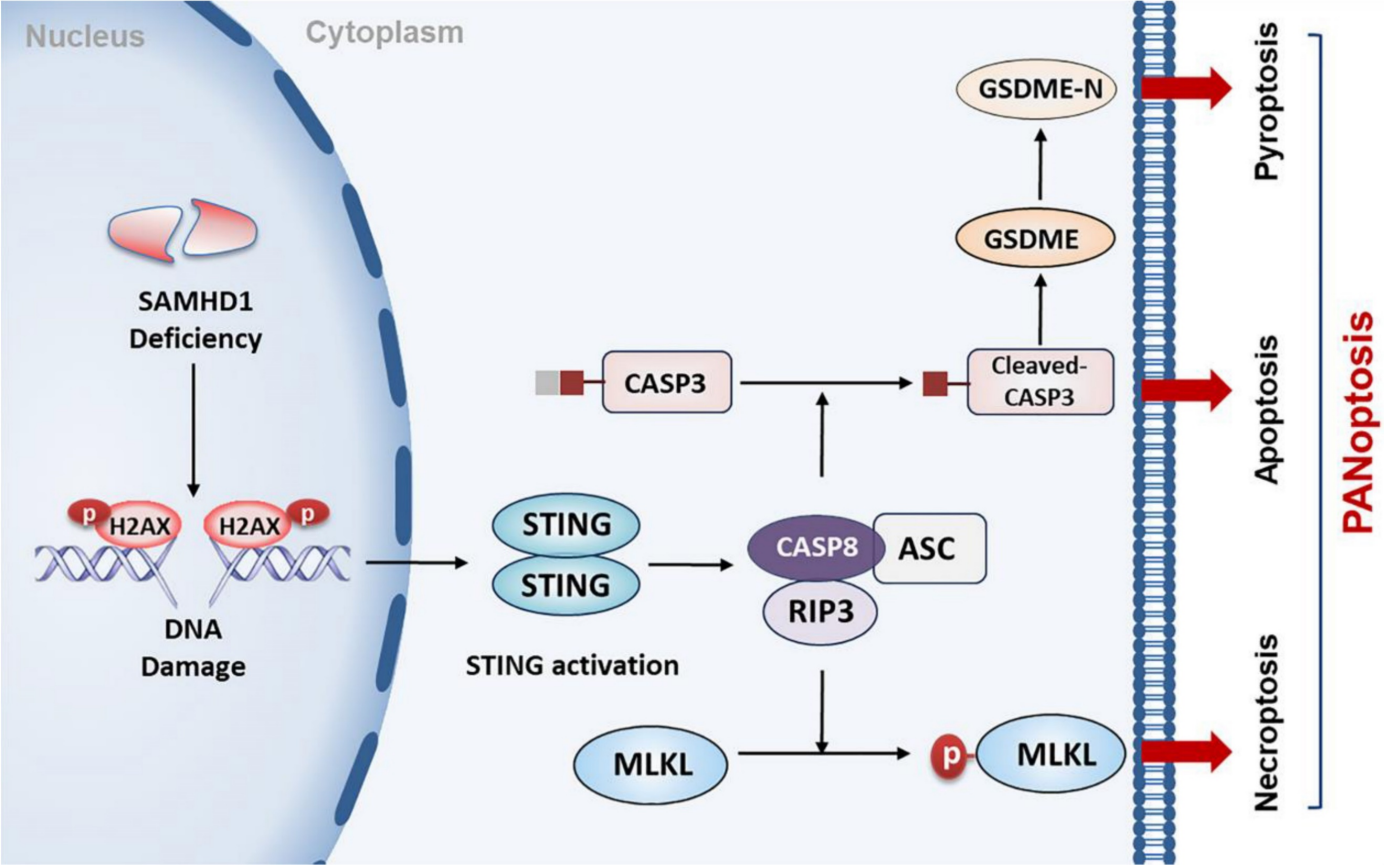
** A proposed model of STING-mediated PANoptosis in DLBCL.** SAMHD1 deficiency induced DNA damage to promote STING activation. Activation of STING led to the formation of CASP8/RIPK3/ASC complex, further activating MLKL, CASP3, and GSDME to induce PANoptosis. Specifically, MLKL phosphorylation induced necroptosis. CASP3 cleavage not only induced apoptosis but also cleaved GSDME to induce pyroptosis.

DLBCL, the most common type of lymphoma, is characterized by high levels of genomic instability. It represents a hugely unmet medical need due to our poor understanding of disease development and the lack of good therapeutic targets. Here, we report, for the first time, that STING activation is facilitated by SAMHD1 deficiency and further restricts tumor growth by inducing PANoptosis in DLBCL cells (**Figure 8**). Additionally, we demonstrate the functions of DMXAA in enhancing the efficacy of anti-PD-L1 treatment, contributing to a potential strategy for DLBCL patients.

In our study, we found that a subset of DLBCL patients with SAMHD1-positive expression was associated with poor prognosis, consistent with the promoting effects of SAMHD1 on tumor growth. SAMHD1 functions in dNTPase-dependent and independent manners 8, 10. The dNTPase activity of SAMHD1 is essential for restricting virus replication and regulating dNTP pools 47, 48. However, SAMHD1 overexpression showed no correlation with viral infections in DLBCL patients. According to the enrichment analysis of RNA-seq, we considered that the functional alterations of SAMHD1 might be attributed to cell cycle checkpoints, especially the G2/M DNA damage checkpoint. In particular, cyclin-dependent kinase 1 (CDK1)-induced SAMHD1 phosphorylation might act as a switch that conversed dNTPase-dependent to independent manners, further recruiting nucleic acid exonucleases to restart the stalled DNA double-stranded replication 10. Therefore, future studies will focus on the post-transcriptional modifications of SAMHD1 in DLBCL. Additional study centers will be included to verify the features of SAMHD1 in DLBCL patients since single-center statistics are limited for characterizing DLBCL as a whole disease.

SAMHD1 has been identified as a negative regulator of innate immunity, as SAMHD1 overexpression prevents the activation of the DNA-sensing pathway 10, 49. Our data established the crosstalk between SAMHD1 and the cGAS-STING axis in DLBCL. Specifically, SAMHD1 deficiency induced DNA damage and cytosolic dsDNA accumulation, which facilitated activating the cGAS-STING pathway 50, 51. Of note, the protein expression of STING, rather than cGAS, was increased with SAMHD1 deficiency. A recent study demonstrated that damage repair proteins directly interacted with STING in the process of DNA damage 52. Therefore, STING activation could be independent of cGAS catalysis. Additionally, our results suggested the specific functions of STING induced by SAMHD1 deficiency. It was well known that STING was associated with cell death regulation and cytokine production 53-55. Necroptosis, caspase-dependent apoptosis, and pyroptosis were mediated by STING in SAMHD1-deficient DLBCL cells. Caspase activation led to cGAS and IRF3 cleavage, further inhibiting cGAS expression and IFN secretion 56. Besides, down-regulation of SAMHD1 promoted NF-κB expression to compensate for the silencing of the IFN pathway 38. Given the importance of cytokines in tumor immune response, future studies will explore the role of STING on cytokine secretion in a natural tumor setting.

So for, the mechanisms of STING-mediated cell death are undefined in DLBCL. Another important exploration in our study was that STING activation inhibited DLBCL tumor growth by inducing PANoptosis. PANoptosis is a newly discovered PCD with features of necroptosis, apoptosis, and pyroptosis 25, 57. Increasing evidence highlights the importance of PANoptosis in human cancers, especially in restricting tumorigenesis, regulating immune response, and enhancing chemosensitivity 27, 58, 59. Constructing a multi-protein complex, known as PANoptosome, is essential for inducing PANoptosis 24. A recent study illustrated that CASP8/RIPK3/ASC multi-protein complex might work as PANoptosome to activate cell death effectors, which was regulated by NF-κB and tumor necrosis factor-α (TNF-α) 38. Complex formation further provides conditions for the interactions of different cell death pathways. Necroptosis is a regulated necrosis that requires the activation of RIPK3 and MLKL 60. As a kind of gasdermin-mediated programmed necrosis, pyroptotic cell death relies on caspase-dependent gasdermin cleavage 61. Consistent with previous studies, STING activation promotes the formation of CASP8/RIPK3/ASC complex in DLBCL cells, further inducing PANoptosis by activating MLKL, CASP3, and GSDME. These data provide evidence for applying STING agonists in anti-DLBCL treatment.

Several kinds of STING agonists are in clinical trials for solid tumors, such as non-small cell lung carcinoma (NSCLC) and melanoma 18. Our study highlighted the anti-tumor effects of STING agonist DMXAA in DLBCL. DMXAA monotherapy suppressed cell viability by inducing cell death. In addition, STING activation may enhance the efficacy of immunotherapy in human cancers 62. Specifically, STING agonists boost anti-tumor immune responses by activating dendritic cells (DCs), natural killer (NK) cells, and IFN-β signaling 62-64. Although PD-1/PD-L1 immunotherapy has emerged as a promising strategy for DLBCL patients, inadequate treatment responses are presented 65, 66. We then established the inhibitory effect of BMS1166 on cell proliferation in DLBCL. However, the functions of BMS1166 might be secondary to other non-specific toxic effects 67. A recent study identifies that activation of STING facilitates overcoming anti-PD-L1 resistance 68. The drug combination study further supported that targeted activation of STING enhances the efficacy of PD-L1 blockade in DLBCL. On the one hand, STING activation promoted the expression of PD-L1 proteins in PD-L1^low^ GCB-like DLBCL cells, which might enhance the sensitivity to PD-L1 blockade 45. On the other hand, combination regimens contributed to reducing drug dosage, which was vital for improving the safety of anti-DLBCL treatment. Therefore, STING agonists may emerge as a potential strategy for DLBCL patients, especially those with low levels of PD-L1.

## Conclusion

In summary, our study highlights the anti-tumor effects of STING in DLBCL. STING activation responds to SAMHD1 deficiency-induced DNA damage and further induces PANoptosis to suppress tumor growth. Combination of STING agonist and PD-L1 inhibitor enhances the efficacy of PD-L1 blockade in DLBCL, especially in PD-L1^low^ GCB-like DLBCL. These findings provide insights into improving the efficacy of anti-DLBCL treatments.

## Supplementary Material

Supplementary figures and tables.

## Figures and Tables

**Table 1:** Correlation between SAMHD1 protein expression and clinicopathologic parameters of DLBCL patients.

Variables	Number of Patients	SAMHD1 expression		*p* value
		Positive	Negative	
**Age (years)**				
≤ 60	50	30 (60.0%)	20 (40.0%)	1.000
> 60	50	30 (60.0%)	20 (40.0%)	
**Gender**				
Male	52	31 (59.6%)	21 (40.4%)	0.935
Female	48	29 (60.4%)	19 (39.6%)	
**Subtype**				
GCB	40	20 (50.0%)	20 (50.0%)	0.248
ABC	60	37 (61.7%)	23 (38.3%)	
**Ann Arbor stage**				
I or II	37	26 (70.3%)	11 (29.7%)	0.108
III or IV	63	34 (54.0%)	29 (46.0%)	
**B symptoms**				
Present	19	7 (36.8%)	12 (63.2%)	0.022^*^
Absent	81	53 (65.4%)	28 (34.6%)	
**Serum LDH levels**				
Normal (LDH-ratio ≤ 1)	53	38 (71.7%)	15 (28.3%)	0.011^*^
Elevation (LDH-ratio > 1)	47	22 (46.8%)	25 (53.2%)	
**ENI**				
Involved site (≤ 1)	24	16 (66.7%)	8 (33.3%)	0.131
Involved site (> 1)	50	24 (48.0%)	26 (52.0%)	
**IPI Score**				
0-2	50	33 (66.0%)	17 (34.0%)	0.221
3-5	50	27 (54.0%)	23 (46.0%)	
**EBV infection**				
Positive	9	5 (55.6%)	4 (44.4%)	0.821
Negative	37	19 (51.4%)	18 (48.6%)	
**HBV infection**				
Positive	27	17 (63.0%)	10 (37.0%)	0.684
Negative	60	35 (58.3%)	25 (41.7%)	
**Treatment response after six cycles of R-CHOP**				
CR and PR	9	8 (88.9%)	1 (11.1%)	0.505
PD	5	3 (60.0%)	2 (40.0%)	

*GCB: Germinal center B-cell; ABC: Activated B-cell; LDH: Lactate dehydrogenase; IPI: International Prognostic Index. B symptoms refer to the fever of unknown cause >38℃ for three consecutive days, night sweats, or weight loss >10% within six months. Extranodal involvement (ENI) excludes testicle, central nervous system and bone marrow due to the significant impact of survival. EBV: Epstein-Barr virus. HBV: Hepatitis B virus. R-CHOP: Rituximab, cyclophosphamide, doxorubicin, vincristine, oral prednisone. CR: Complete remission. PR: Partial remission. PD: Progressive disease
