# Supplementary material for: Activation of STING by SAMHD1 Deficiency Promotes PANoptosis and Enhances Efficacy of PD-L1 Blockade in Diffuse Large B-cell Lymphoma
Source: Int J Biol Sci. 2023 Aug 28;19(14):4627–43. doi: 10.7150/ijbs.85236 (PMC10535696; doi:10.7150/ijbs.85236)
Supplement: Supplementary file 1 — Supplementary figures and tables. [file ijbsv19p4627s1.pdf]

Figure S1

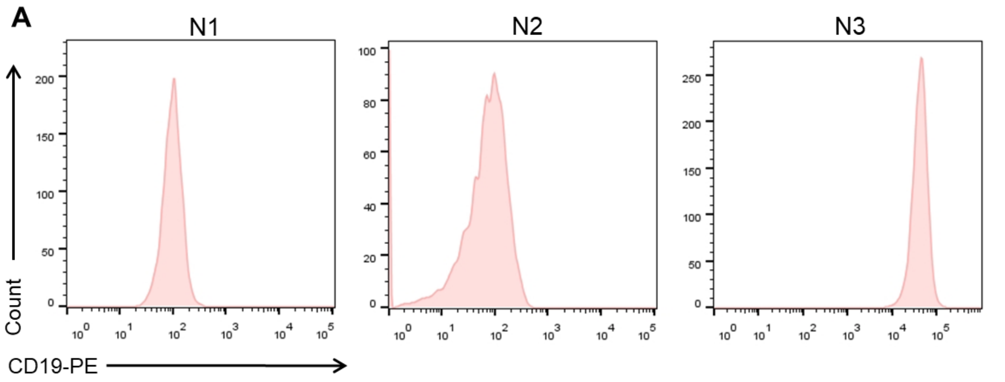

A. Naïve CD19^{+} B-cells from healthy donors (n=3) were separated by CD19^{+} magnetic microbeads. Flow cytometry revealed the enrichment of CD19^{+} B-cells by single peak diagram.

Figure S2

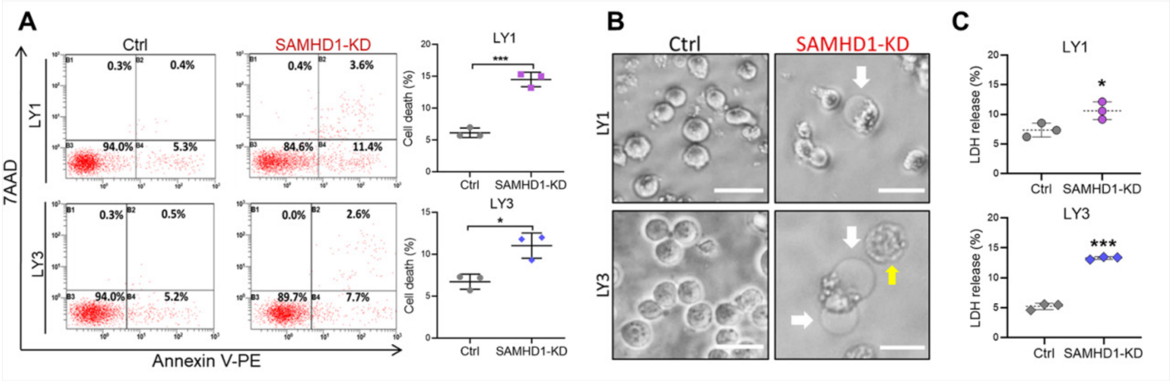

A. Annexin V-PE/7AAD double staining flow cytometry revealed the scatter plots (left) and quantitative apoptosis rates (right) in Ctrl and SAMHD1-KD DLBCL cells. **B.** Microscopic images of Ctrl and SAMHD1-KD DLBCL cells (scale bar=50μm). White arrows indicated cell membrane swelling, while yellow arrows showed cell shrinkage. **C.** LDH release assay showed the supernatant LDH levels of Ctrl and SAMHD1-KD DLBCL cells. Vertical bars indicated mean ± SD. P values came from unpaired two-tailed t-test (**A, C**). *p<0.05, ***p<0.001.

.

Figure S3

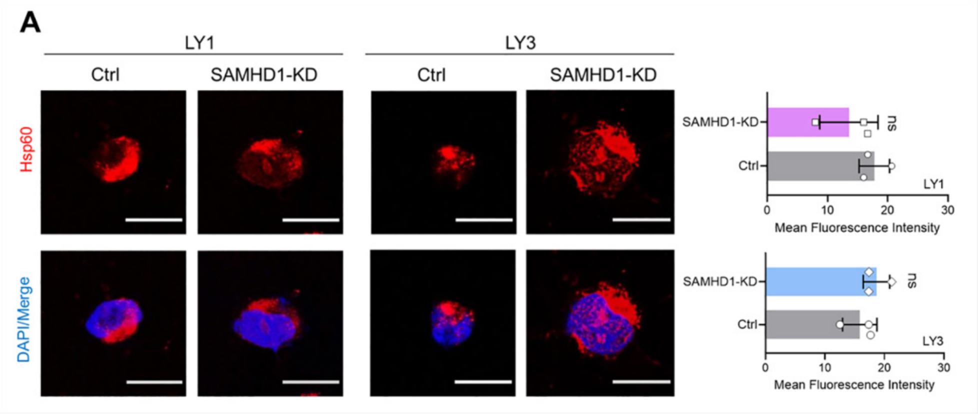

A. Representative fluorescence plots (left) and fluorescence density (right) of Hsp60 in Ctrl and SAMHD1-KD DLBCL cells (scale bar=10μm). Vertical bar indicated mean ± SD. ns= no significance.

Figure S4

A

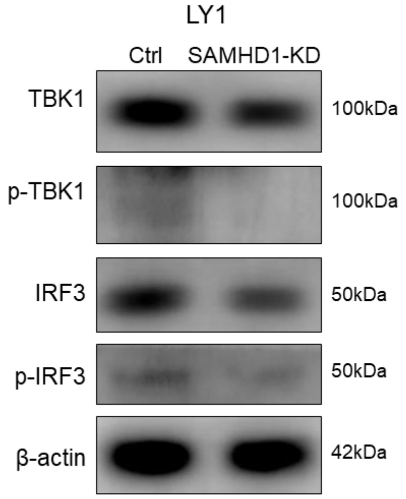

A. Immunoblot revealed the protein levels of TBK1, p-TBK1, IRF3, and p-IRF3 in LY1 cells transfected with Ctrl or SAMHD1-KD.

Figure S5

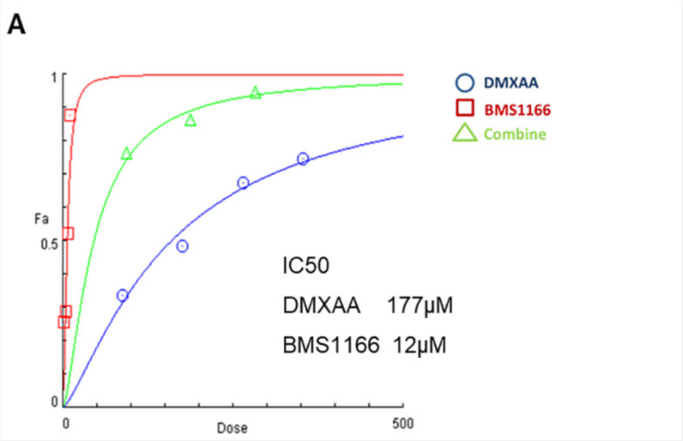

A. The IC50 of DMXAA and BMS1166 in drug combination was calculated according to the dose-effect curve (DMXAA IC50=177μM, BMS1166 IC50=12μM).

Table S1. Treatment regimens and CR rates of 49 DLBCL patients.

Treatment regimens	Number of Patients (n=49)	Patients with CR[1]
3 cycles of R-CHOP[2]	7	2 /7, 28.6%
6 cycles of R-CHOP	14	10/14, 71.4%
6 cycles of CHOP	2	2/2, 100.0%
6 cycles of CHOP + 6 cycles of R-GDP[3]	1	0/1, 0.0%
6 cycles of R-CHOP + 1 cycle of Gemox[4]	2	0/2, 0.0%
5 cycles of BTK inhibitor (Zanubrutinib) + 1 cycle of Rituximab	1	1/1, 100.0%
Others[5]	22	2/22, 9.1%

1. CR: Complete remission.

2. R-CHOP: Rituximab, Cyclophosphamide, Doxorubicin, Vincristine and oral Prednisone.

3. R-GDP: Rituximab, Gemcitabine, Dexamethasone, Cisplatin.

4. Gemox: Gemcitabine, Oxaliplatin.

5. Others: The regimens containing 2 or more second-line regimens, including Cyclophosphamide + Doxorubicin + Vincristine + Prednisone + Etoposide (CHOPE), Methotrexate + Cytarabine (Hyper-CVAD B), Ifosfamide + Carboplatin + Etoposide (ICE), Etoposide + Prednisone + Vincristine + Cyclophosphamide + Doxorubicin (EPOCH), local radiotherapy, allogeneic hematopoietic stem cell transplantation (ASCT), etc.

Table S2. Sequences of lentivirus-mediated knockdown and vector controls.

Target genes	Sequences
SAMHD1-KD sh1#	5'-CCAGTGCTAAACCCAAAGTAT-3'
SAMHD1-KD sh2#	5'-GCAGATGACTACATAGAGATT-3'
Ctrl	hU6-MCS-CBh-gcGFP-IRES-puromycin
LV-Con	Ubi-MCS-3FLAG-CBh-gcGFP-IRES-puromycin

Table S3. Resource and dilution of antibodies applied in IF.

Antibodies	Resource	Dilution
Mouse anti-dsDNA	Abcam, ab27156, Cambridge, MA, USA	1:100
Rabbit anti-phospho-histone H2A.X	Cell Signaling Technology, 9718, MA, USA	1:400
Rabbit anti-HSP60	Proteintech, 15282-1-AP, IL, USA	1:200
Goat anti-Rabbit IgG (H+L) Cross-Adsorbed Secondary Antibody	Thermo Fisher, A-11012, CA, USA	1:400
Goat anti-Mouse IgG (H+L) Cross-Adsorbed Secondary Antibody	Thermo Fisher, A-11029, CA, USA	1:400

Table S4. Resource and dilution of antibodies applied in immunoblot analysis.

Antibodies	Resource	Dilution
Rabbit anti-SAMHD1	Proteintech, 12586-1-AP, IL, USA	1:1000
Rabbit anti-GSDME	Proteintech, 13075-1-AP, IL, USA	1:1000
Mouse anti-PD-L1	Proteintech, 66248-1-Ig, IL, USA	1:1000
Rabbit anti-RIPK3	Proteintech, 17563-1-AP, IL, USA	1:1000
Mouse anti-Caspase8/p43/p18	Proteintech, 66093-1-Ig, IL, USA	1:1000
Rabbit anti-cGAS	Cell Signaling Technology, 15102, MA, USA	1:1000
Rabbit anti-STING	Cell Signaling Technology, 13647, MA, USA	1:1000
Rabbit anti-ASC/TMS1	Cell Signaling Technology, 13833, MA, USA	1:1000
Rabbit anti-Caspase3	Cell Signaling Technology, 14220, MA, USA	1:1000
Rabbit anti-Cleaved-Caspase3	Cell Signaling Technology, 9664, MA, USA	1:1000
Rabbit anti-MLKL	Cell Signaling Technology, 14993, MA, USA	1:1000
Rabbit anti-p-MLKL	Abcam, ab187019, Cambridge, MA, USA	1:1000
Rabbit anti-p-RIPK3	Abcam, ab209384, Cambridge, MA, USA	1:1000
Rabbit anti-N-GSDME	Abcam, ab215191, Cambridge, MA, USA	1:1000
Rabbit anti-TBK1	Abcam, ab40676, Cambridge, MA, USA	1:1000
Rabbit anti-p-TBK1	Abcam, ab109272, Cambridge, MA, USA	1:1000
Rabbit anti-IRF3	Abcam, ab68481, Cambridge, MA, USA	1:1000
Rabbit anti-p-IRF3	Abcam, ab76493, Cambridge, MA, USA	1:1000
Mouse anti-β-actin	Abcam, ab8226, Cambridge, MA, USA	1:1000
Mouse anti-GAPDH	Zhong Shan-Golden Bridge, TA-08, Beijing, China	1:1000
Goat anti-rabbit IgG horseradish peroxidase-linked secondary antibody	Zhong Shan-Golden Bridge, ZB-5301, Beijing, China	1:5000
Goat anti-mouse IgG horseradish peroxidase-linked secondary antibody	Zhong Shan-Golden Bridge, ZB-5305, Beijing, China	1:5000
